# Compensation of Temperature-Induced Bias Drift in Hemispherical Resonator Gyroscopes: An Inherent Data-Driven Architecture

**DOI:** 10.3390/mi16040357

**Published:** 2025-03-21

**Authors:** Xiaocong Zhou, Jiaqiang Wen, Shasha Han, Chong Li

**Affiliations:** School of Engineering, Ocean University of China, Qingdao 266404, China; 17860819682@163.com (X.Z.); wenjiaqiang@stu.ouc.edu.cn (J.W.); hanshasha@stu.ouc.edu.cn (S.H.)

**Keywords:** hemispherical resonator gyroscope, zero-rate output bias, hysteresis phenomenon, KAN neural network, real-time compensation model

## Abstract

To address the bias drift problem and hysteresis phenomenon of hemispherical resonator gyroscope (HRG) under temperature change, a temperature drift compensation method based on internal parameters is proposed. The influence model of zero-rate output bias is established with the parameters such as resonance frequency, driving signal amplitude and quadrature suppression voltage amplitude during HRG operation. The temperature cycle experiment is carried out in the range of −20 to 60 °C, and the relationship between internal parameters and working temperature is revealed. Using KAN neural network combined with time series data as input features, a real-time compensation model is designed to effectively improve the prediction accuracy of hysteresis phenomenon. The experimental results show that the model significantly reduces the output stability performance of HRG, from 0.022°/h to 0.013°/h, and the stability decreases from 1.1392°/h to 0.0651°/h, which improves the stability and reliability of HRG.

## 1. Introduction

HRG exhibits high precision, reliability, and longevity [1,2], making it the most promising gyroscope for inertial navigation systems [3,4]. Its operational principle [5,6] relies on detecting the base rotation through Coriolis-induced precession of the radial vibration standing waves in a hemispherical resonator. When the base rotates, the vibration mode shifts proportionally to the rotation angle [7]. Extensively implemented in advanced systems like deep-space probes [8], naval vessels [9], satellites [10], and missile guidance [11], HRG performs critical navigation, positioning, and attitude control functions [12].

HRG performance indicators require enhancement for cutting-edge applications, particularly demanding stable zero-rate output bias under significant temperature variations [13,14,15,16]. Although the quartz glass resonator exhibits low thermal expansion, substantial temperature changes still cause zero-point drift [17], limiting HRG’s application in extreme environments. Effective temperature drift modeling and compensation [18,19] remain crucial for improving HRG measurement accuracy and expanding its applications.

Enhancing HRG temperature performance primarily involves two strategies: optimizing manufacturing/assembly processes and studying temperature compensation mechanisms. UC Irvine’s Shkel team [20] developed a circular-level micro glass blowing process to create fused silica resonators with a Q-factor exceeding 1 million. University of Michigan’s Najafi team [21] used a vacuum negative-pressure technology to fabricate micro-hemispherical gyros with Q-factors over 100 million. Wang Hua et al. [22] (Harbin Institute of Technology) established a dynamic slip model based on fractal contact theory to guide contact parameter optimization. Yuan Lishan’s team [23] (Harbin Institute of Technology) modeled energy dissipation mechanisms, clarifying how mass defects affect Q-factors. Gu Xibing’s team [24] (Beijing Information Science and Technology University) optimized the structural parameters of all-metal resonators, reducing frequency splitting to 14 Hz. These methods mitigate temperature effects by improving Q-factors and minimizing assembly errors. However, they lack specificity, and struggle to eliminate circuit-related temperature errors.

Traditional methods for error mechanism modeling and compensation include polynomial fitting models [25], autoregressive multivariate models [26], and neural network models [27,28]. Among improved approaches, the dual-gyro self-calibrating CVG system proposed by Northrop Company [29] detects input information through time-sharing operation modes. The CETC 26th Institute team applied temperature compensation circuits [30] to superimpose real-time compensation signals, but predefined temperature compensation coefficients are required. The Tianjin Navigation Institute team introduced a compensation method combining gray models and RBF neural networks [31], reducing errors by an order of magnitude compared to polynomial models. The Beijing University of Aeronautics and Astronautics team developed a dynamic non-linear RIRG error model integrated with particle swarm optimization [32], effectively suppressing bias drift. Although these methods can effectively offset the drift error of the gyro, most fail to account for hysteresis effects and other dynamic thermal factors.

The contributions of this work are summarized as follows: (1) an intelligent compensation method is proposed, utilizing internal gyroscope parameters (e.g., resonant frequency, driving signal amplitude, and AC suppression signal) to predict zero-rate output bias. (2) A temperature drift prediction model is developed using Kolmogorov–Arnold Networks (KAN) [33], leveraging the gyroscope’s internal error mechanism and deep learning’s non-linear capabilities. (3) A time series prediction strategy is introduced to address temperature-induced hysteresis, incorporating the current time output as a feature for the next moment. Experimental results demonstrate enhanced temperature bias stability in the hemispherical gyroscope.

## 2. Operation Mechanism of Hemispherical Resonant Gyroscope

**Figure 1:**
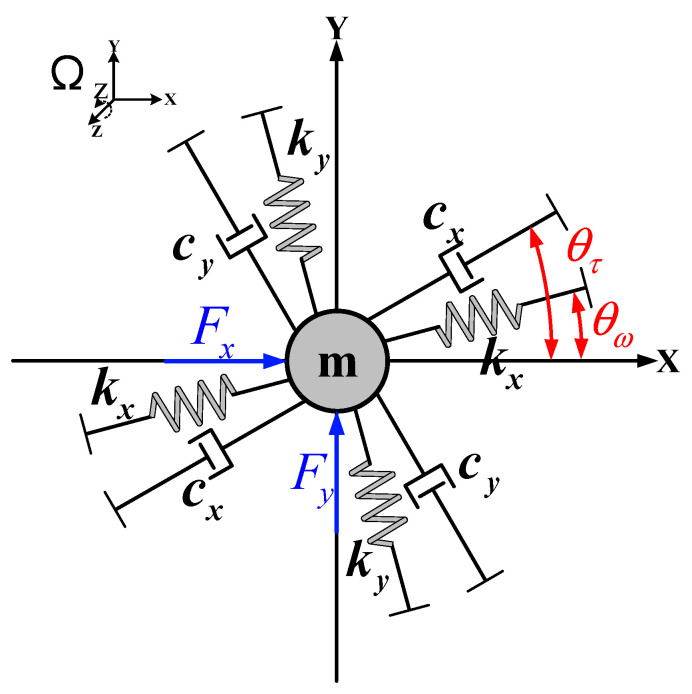
HRG engineering dynamic model.

The core principle of the HRG lies in the resonator’s vibration, which is induced by the Coriolis force, enabling precise measurement of the rotational angular rate. One of its engineered dynamic models is shown in Figure 1, and the main dynamic characteristics of HRG can be further represented by differential equations:

(1) $$\left\{\begin{array}{l} m\ddot{x}+c_{x}\dot{x}+k_{x}x+c_{xy}\dot{y}+k_{xy}y=F_{x}+2m\lambda \Omega_{z}\dot{y}\\ m\ddot{y}+c_{y}\dot{y}+k_{y}y+c_{xy}\dot{x}+k_{xy}x=F_{y}-2m\lambda \Omega_{z}\dot{x} \end{array}\right.$$

In Figure 1 and Equation (1), x, y represent the vibration displacements of the gyroscope in the X and Y vibration modes, respectively. m is the equivalent mass of the resonator. $c_{x}$, $c_{y}$ are the damping coefficients of the two modes. $k_{x}$, $k_{y}$ are the stiffness coefficients of the two modes. $\theta_{\tau }$, $\theta_{\omega }$ are the damping and stiffness azimuths separated from the vibration modes. $c_{xy}$, $k_{xy}$ are the damping coupling coefficient and stiffness coupling coefficient caused by the azimuth mismatch shown in the figure, respectively. $F_{x}$, $F_{y}$ are the driving signals of the two vibration modes. $\Omega_{z}$ represents the angular rate input on the z-axis. λ represents the angular gain coefficient. $2m\lambda \Omega_{z}\dot{x}(t)$ and $2m\lambda \Omega_{z}\dot{y}(t)$ are the Coriolis forces acting on the two modes.

Under the force-balance modulation scheme, a driving force $F_{x}=F_{0}cos(\omega_{x}t)$ can be applied to the X-mode of the system, where $F_{0}$ represents the amplitude information of the input signal, and $\omega_{x}$ represents the frequency of the input signal. And the driving force of the Y-mode is set as $F_{y}=0$. Also, the Coriolis force $2m\lambda \Omega_{z}\dot{y}(t)$ of the Y-mode on the X-mode is neglected. Therefore, the time-domain response solutions of the X-mode and Y-mode are:

(2) $$\begin{array}{l} x(t)=\frac{Q_{x}F_{0}}{m\omega_{x}^{2}}\cdot sin(\omega_{x}t)\\ y(t)=\frac{Q_{x}F_{0}}{m\omega_{x}^{2}}\cdot \frac{\left(2\lambda \Omega_{z}+\frac{c_{xy}}{m})\cdot sin(\omega_{x}t+\phi )-\frac{k_{xy}}{m\omega_{x}}\cdot Q_{x}\cdot cos(\omega_{x}t+\phi \right)}{\sqrt{{\left(\Delta \omega \right)}^{2}+{\left[\omega_{y}\omega_{x}/Q_{y}(\omega_{y}+\omega_{x})\right]}^{2}}}\\ \phi =\frac{\pi }{2}-tan^{-1}(\frac{\omega_{y}/Q_{y}}{2\Delta \omega }) \end{array}$$

In the above equations, $\omega_{x}=\sqrt{k_{x}/m}$ and $\omega_{y}=\sqrt{k_{y}/m}$ represent the natural frequencies of the two modes, respectively, $\Delta \omega =\omega_{x}-\omega_{y}$ represents the frequency splitting of the two modes, $Q_{x}=m\omega_{x}/c_{x}$ and $Q_{y}=m\omega_{y}/c_{y}$ represent the quality factors of the two modes.

## 3. Mapping Relationship Between Gyroscope Zero-Position Output and Temperature Change

### 3.1. Single Point Temperature Change and Gyro Bias

Ideally, the HRG’s output reflects the Coriolis force magnitude, yielding zero output at zero input angular rate $\Omega_{z}$. However, in practice, geometric deviations, material inhomogeneity, manufacturing asymmetry, and internal stresses alter the resonator’s vibration modes [34], causing drive mode input to couple into detection mode. This results in a detectable output at zero input, known as the zero-rate output bias, as shown in Equation (2).

The bias characteristics of HRG are influenced by various factors, among which the change in ambient temperature T significantly affects the zero-rate output bias. Temperature variations can alter the physical properties of the materials inside the gyroscope, such as the elastic modulus E, Poisson’s ratio μ, density ρ, the thickness of the hemispherical shell h, and the shell radius r. These changes lead to alterations in the geometric shape of the resonator, thereby affecting the resonant frequency ω, as shown in Equation (3) [35].

(3) ω(T)=1.5127⋅h(T)r2(T)⋅E(T)(1+μ(T))ρ(T)12

Meanwhile, the drift of damping coefficient $c_{x}$, $c_{y}$ and stiffness coefficient $k_{x}$, $k_{y}$ alters the zero-bias output, transforming Equation (2) into Equation (4), which indicates temperature error effects, and can be used to compensate for environmental temperature impacts on HRG, enhancing its temperature stability and measurement accuracy.

(4) $$\begin{array}{c} y(t)=\frac{Q_{x}(T)Q_{y}(T)F_{0}}{m^{2}\omega_{x}^{3}(T)}\cdot (c_{xy}(T)sin(\omega_{x}(T)t+\phi )\\ -\frac{k_{xy}(T)}{\omega_{x}(T)}\cdot Q_{x}(T)\cdot cos(\omega_{x}(T)t+\phi )) \end{array}$$

### 3.2. Hysteresis Effect of Temperature Sequence on Gyro Zero-Rate Output Bias

Temperature has a significant impact on the zero-rate output bias of HRG, yet the relationship between them is not a simple one-to-one mapping. When describing the relationship between the zero-rate output bias of HRG and temperature, there is an important non-linear phenomenon: the hysteresis effect.

**Figure 2:**
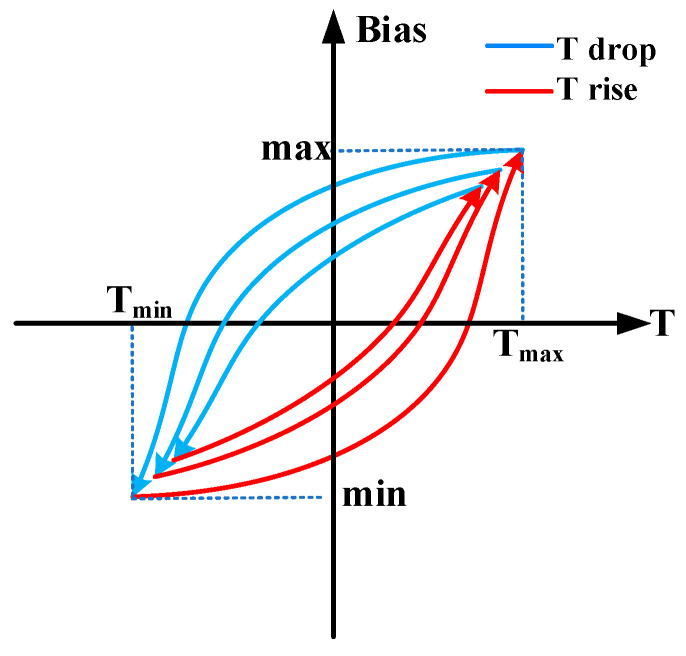
Zero-rate output bias–temperature hysteresis curve.

The hysteresis effect describes how the zero-rate output bias of a gyroscope is influenced not only by the current temperature but also by past temperature trends, showing a historical dependence on temperature. This is particularly noticeable in temperature cycling experiments, where the bias during heating and cooling differs even at the same temperature point. As illustrated in Figure 2, the distinct change trends during temperature increase and decrease define the hysteresis effect. This means that even when the temperature returns to its initial value, the gyroscope’s bias may not revert to its original state.

## 4. Research on Internal Parameter Drive and Zero-Rate Output Bias Compensation Method of Gyroscope

### 4.1. Temperature Change and Internal Compensation Parameter Selection

The uncertainty of environmental temperature and the immeasurability of internal temperature are the main challenges in achieving effective temperature compensation. Referring to Equation (3), if the internal temperature changes in the gyroscope are consistent with the resonant frequency, the resonant frequency variation can be considered as a representation of temperature change. However, the interaction between resonant frequency and temperature is complex, making it difficult to establish an accurate theoretical model. Therefore, an experimental approach [35] is more practical. By fitting experimental data to establish an empirical relationship between resonant frequency and internal temperature, the reliability and effectiveness of the model can be ensured.
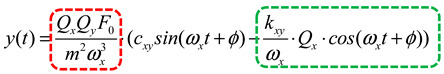
(5)

In addition to the resonant frequency, other parameters of the HRG, such as the amplitude of the drive signal and the amplitude of the quadrature suppression voltage, also vary with temperature. As expressed in Equation (5), this is the zero-rate bias output formula of the gyroscope. The red-colored terms correspond to factors affecting the bias: resonant frequency and drive amplitude, while the green-colored terms represent stiffness coupling coefficients and resonant frequency influencing the bias, thereby characterizing the impact of the gyroscope’s quadrature component. Therefore, when designing a temperature drift compensation strategy, it is necessary to comprehensively consider the temperature dependence of these parameters. Through precise measurement and modeling of multiple parameters, the impact of temperature changes on gyroscope performance can be more comprehensively predicted, providing a scientific basis for achieving more precise zero-rate output bias compensation and enhancing the temperature adaptability and overall accuracy of the HRG.

### 4.2. Look-Up Table Method Based on Gyro Resonant Frequency

In HRG temperature drift compensation, the look-up table (LUT) method is widely used. It reduces environmental sensitivity by leveraging pre-recorded temperature cycle test data, establishing a relationship between temperature T and zero-rate output bias. In practice, the detected environmental temperature is used to find the corresponding bias value for compensation. Since direct measurement of HRG’s internal temperature is challenging, this paper replaces actual temperature with the observable resonant frequency ω, creating a frequency-to-bias correspondence table. During operation, the current resonant frequency is used to identify the corresponding bias value for drift compensation.

### 4.3. Research on Least-Squares Algorithm with Time Series Characteristics

To more accurately describe the effect of temperature changes on the zero-rate output bias of the HRG, the least-squares method is employed to estimate the coefficients of the temperature error model. This method minimizes the sum of squared errors to find the best-fitting parameters, thereby establishing a mathematical model that describes the relationship between temperature and bias drift. In this paper, the resonant frequency is used as a substitute for the internal operating temperature of the HRG to construct the drift model. However, the influence of temperature on the gyroscope’s zero-rate output bias exhibits a certain temporal correlation, rather than being merely an instantaneous effect. Therefore, other independent variables such as {f, Δf, $f^{2}$, $\Delta f^{2}$, Δf⋅f, $f\cdot \sqrt{f}$, $\Delta f\cdot \sqrt{f}$, $\Delta f\cdot \sqrt{f}\cdot f$} are considered to be constructed based on the resonant frequency. Here, f is the resonant frequency, △f is the difference between the current resonant frequency and the resonant frequency of the previous moment, and $\sqrt{f}$ is the square root of the resonant frequency. The established multiple regression model is described as follows:

(6) $$\begin{array}{ll} v_{o} & =a_{0}+a_{1}\cdot f+a_{2}\cdot \Delta f+a_{3}\cdot \sqrt{f}+a_{4}\cdot f^{2}\\ & +a_{5}\cdot \Delta f^{2}+a_{6}\cdot \Delta f\cdot f+a_{7}\cdot f\cdot \sqrt{f}\\ & +a_{8}\cdot \Delta f\cdot \sqrt{f}+a_{9}\cdot \Delta f\cdot \sqrt{f}\cdot f \end{array}$$

$v_{0}$ represents the zero-rate output bias compensation value of the HRG at the current moment. $a_{i}$ is the coefficient of each term in the model, and its magnitude is determined according to the least-squares method.

### 4.4. Intelligent Compensation Method Based on Internal Parameter Drive

The two methods compensate for the deterministic biases caused by temperature changes to a certain extent. However, due to the strong non-linearity of the HRG’s zero-rate output bias and the presence of the hysteresis effect, the final compensation results deviate significantly from the expected outcomes.

Against this backdrop, the KAN network model, with its unique structure and advantages, offers a new solution for the research on zero-rate output bias compensation of HRG. KAN is a new type of deep learning model that has demonstrated great potential and advantages in fields such as pattern recognition, time series prediction, and control systems. KAN is constructed based on the KA theorem, which states that any continuous function $f(x_{1},\cdots ,x_{n})$, defined on a closed interval, can be approximated by a combination of a finite number of one-dimensional functions. Its unique mathematical expression is as follows:

(7) $$\begin{array}{ll} f(x) & =f(x_{1},x_{2},\cdots ,x_{n})\\ & =\sum_{q=1}^{2n+1}\Phi_{q}(\sum_{p=1}^{n}\phi_{p,q}(x_{p})) \end{array}$$

Among them, $\phi_{q,p}(x_{p})$ is a one-dimensional continuous function that maps a single variable to real numbers; $\Phi_{q}$ is a one-dimensional continuous function that maps the “sum of real numbers” after the mapping by $\phi_{q,p}(x_{p})$ to real numbers.

The core characteristic of the KAN [33] network lies in the fact that its network model has no linear weights at all. Each weight parameter is replaced by a learnable univariate activation function ϕ(x), as shown in Equation (8) [33]. These activation functions are usually parameterized using B-splines. Through the innovative weight replacement and the design of the activation function positions, the performance of the KAN network is significantly improved, making it superior to the traditional MLP in terms of accuracy.

(8) $$\begin{array}{l} \phi (x)=\omega (b(x)+spline(x))\\ b(x)=silu(x)=x/(1+e^{-x})\\ spline(x)=\sum_{i}c_{i}B_{i}x \end{array}$$

**Figure 3:**
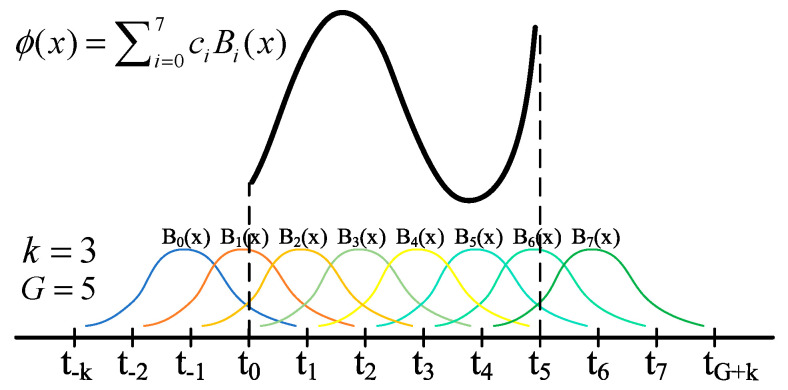
B-spline curve combination.

The learnability of the activation function is manifested in that it is formed by fitting the basis function b(x) and multiple spline functions spline(x), as shown in Figure 3. Here, the parameter $c_{i}$ is the scaling coefficient of each spline function, k represents the order of the spline function, and the number of grid points G determines the shape and coverage of these basis functions. By refining the mesh, the adaptability of the spline functions can be improved, enabling them to fit complex function shapes more precisely. This design of the non-linear kernel function and the adjustable fineness of the nodes endows it with higher flexibility when dealing with the task of predicting the gyroscope’s zero-rate output bias.

Based on the KAN network, this paper innovatively proposes a temperature drift compensation method driven by the internal parameters of the gyroscope. The aim is to construct a temperature drift compensation model that can make predictions based on the visualizable parameters during the gyroscope’s operation, so as to enhance the stability of the output of the gyroscope in a temperature-varying environment.

**Figure 4:**
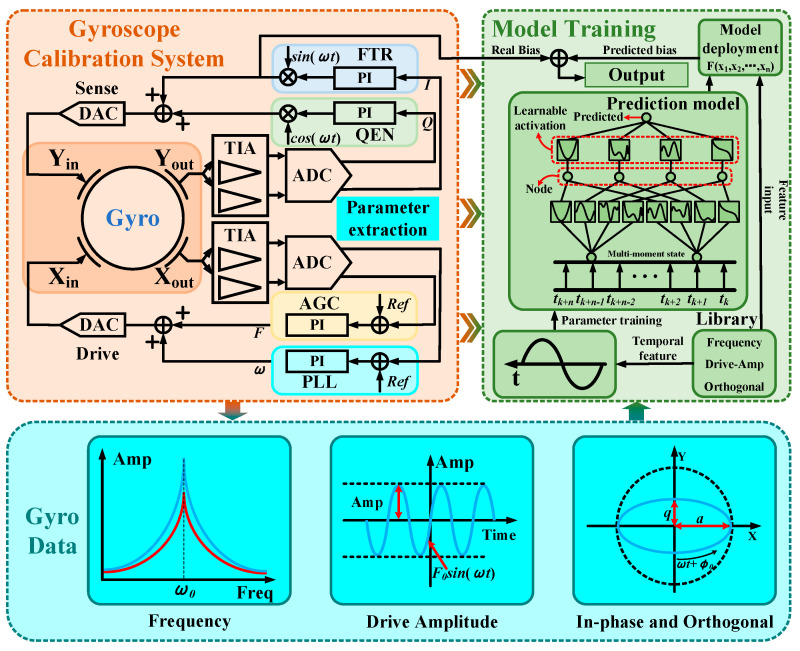
Architecture of zero-rate output bias compensation scheme driven by internal parameters of gyroscope based on KAN network.

Figure 4 shows the architecture diagram of this compensation scheme. The orange part represents the gyroscope calibration system model used for parameter extraction. The core function of this model is to provide an excitation signal for the HRG and perform closed-loop control on its output signal. The closed-loop control strategy consists of four key parts: frequency control, amplitude control, quadrature control, and force balance control. Using the principle of negative feedback suppression, the signal output by the ADC is collected as the feedback quantity, and the controller generates a suppression force to keep the control quantity stable at all times. The control quantity refers to the internal parameters during the operation of the HRG, which are collected in real-time by four control loops, such as the resonant frequency, drive amplitude, quadrature and in-phase suppression voltages, represented by the blue part. Among them, the resonant frequency, drive amplitude, and quadrature suppression voltage are temperature-related, and the in-phase suppression voltage is an important representation of angular rate information.

The four collected data items serve as input features and output labels, respectively, providing a basis for parameter fitting for the KAN network model in the green part. Due to the non-linear characteristics and possible hysteresis phenomena in the HRG output, this paper refers to the data input strategy of time series prediction and attempts to comprehensively consider the sample data at multiple time points when training the network. The output at the current moment is used as one of the input features at the next moment, hoping to help capture and predict the time series characteristics of the HRG output, thereby improving the accuracy and efficiency of zero-rate output bias compensation.

The combination of the KAN network method and gyroscope temperature drift compensation provides a brand-new solution. The structure of the KAN network allows for the interpretation and formula-based understanding of the learning process and results of gyroscope output prediction. This interpretability is crucial for monitoring and verifying the model’s effectiveness and facilitates its application in actual prototypes. By applying the KAN network to the temperature drift compensation problem of HRG, not only can high-precision error compensation be achieved, the behavior patterns under different environmental conditions can also be captured through its deep structure, providing an accurate and reliable compensation method. The implementation of this method is expected to significantly improve the temperature adaptability and overall accuracy of the HRG, ensuring its stability and reliability under different temperature environmental conditions.

## 5. Experimental Results and Analysis

### 5.1. Experimental Design and Data Collection

**Figure 5:**
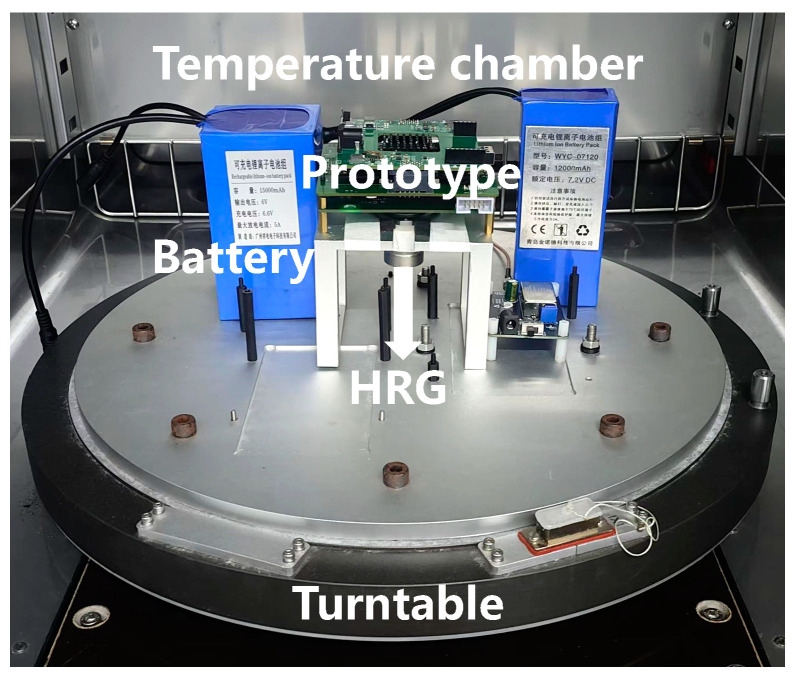
Temperature experimental environment.

A continuous temperature experiment for the HRG is designed to analyze the relationship between the internal parameters of the gyroscope and the actual temperature. The experimental environment setup is shown in Figure 5. In the experiment, a temperature chamber is used to control the environmental temperature. Temperature adjustment instructions are set for the temperature chamber in advance. After the temperature inside the chamber reaches the set initial temperature, the gyroscope is powered on. It is kept at a constant temperature for 2 h to make the internal temperature of the gyroscope consistent with the environmental temperature, ensuring that the gyroscope is in a working state and the accuracy of the internal temperature measurement.

The designed temperature range of the experimental environment is from −20 °C to 60 °C. The temperature chamber is used to control the heating and cooling rates of the environment at 0.5 °C/min. With an interval of 10 °C, the temperature is maintained at −20 °C, −10 °C, 0 °C, 10 °C, 20 °C, 30 °C, 40 °C, 50 °C, and 60 °C for 1 h, respectively. The temperature cycle is repeated three times, and data such as the resonant frequency, drive amplitude, quadrature suppression voltage, and angular rate output of the HRG are recorded.

**Figure 6:**
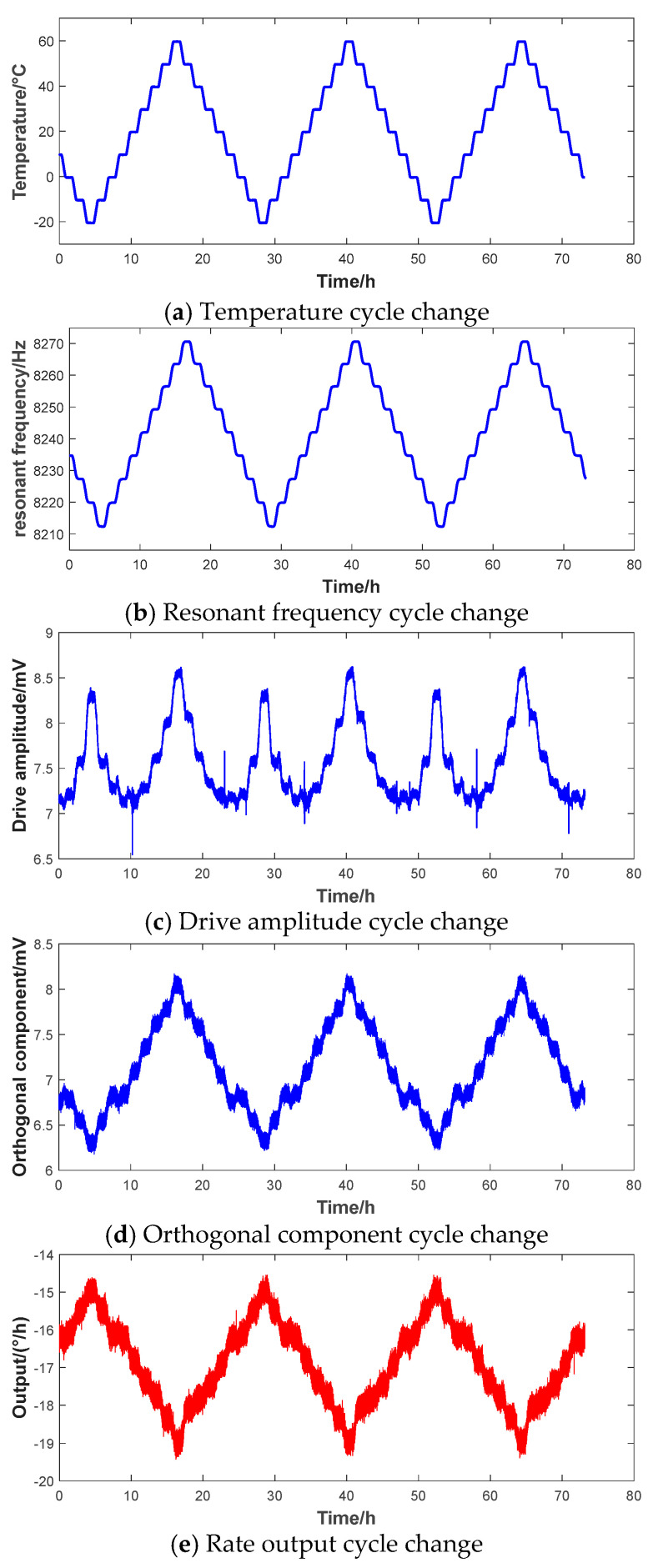
Curves of key parameters of HRG varying with temperature.

Figure 6 shows the variation curves of the environmental temperature, resonant frequency, drive amplitude, quadrature suppression voltage, and the measured angular rate when the HRG is undergoing a temperature cycling experiment. By analyzing these curves, it can be observed that as the temperature fluctuates, there is an obvious correlation among the resonant frequency, drive amplitude, and quadrature suppression voltage. The change in drive amplitude even exhibits obvious non-linear characteristics. Meanwhile, in Figure 6e, the angular rate output, represented in red, shows a trend consistent with the variations in the above three parameters. This further validates the feasibility of the zero-rate output bias compensation method adopted under the influence of internal parameters. The results demonstrate that precise control and adjustment of the internal parameters of the gyroscope contribute to compensating for the temperature drift of the HRG, ensuring its stability and reliability under different temperature conditions.

### 5.2. Scheme Verification and Performance Comparison

Before validating the scheme, it is necessary to test the basic performance of the HRG to evaluate the performance improvement effect of the adopted method. With the help of a turntable, the experiment measures that the bias instability of the HRG in a temperature-varying environment is 0.022°/h, and the bias stability is 1.1392°/h.

**Figure 7:**
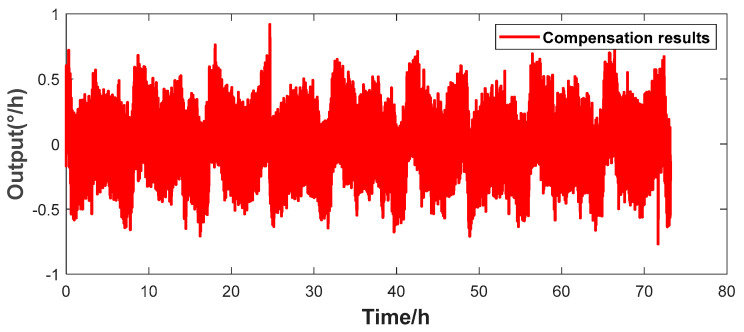
Angular rate output after compensation by the look-up table method.

Figure 6 shows that there is a good linear relationship between the environmental temperature and the resonant frequency. This paper improves the traditional look-up table method. By experimentally simulating the bias drift data of the HRG under different conditions, the real-time resonant frequency is used to replace the temperature and stored in a look-up table. When the gyroscope is in operation, the corresponding zero-rate output bias error value is found in the look-up table, according to the current resonant frequency for correction. The actual compensation effect is shown in Figure 7. The calculated bias instability of the compensation result is 0.0199°/h, and the bias stability is 0.1312°/h.

**Figure 8:**
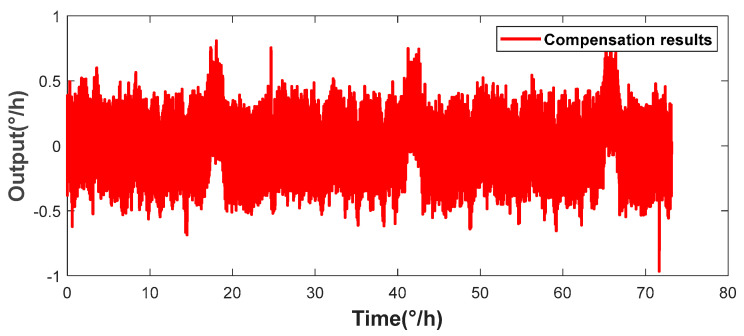
Angular rate output after compensation by the least squares method.

The look-up table method has a certain compensation effect. However, as shown in Figure 8, due to the influence of the hysteresis effect, the zero-rate output bias compensation effects at different times vary greatly, and the resulting impact cannot be ignored. Meanwhile, in the case of long-term operation, errors gradually accumulate, indicating that relying solely on the resonant frequency cannot establish an accurate mapping relationship with the gyroscope’s bias. Therefore, the multiple regression model described in Section 4.3 is used. Based on the pre-collected experimental data, the mean values of the resonant frequency and the bias are calculated every 200 data points and substituted into Equation (6) to match the optimal fitting function coefficients. Figure 8 shows the bias compensation results of the multiple regression model using the optimal coefficients. The calculated bias instability of the gyroscope is 0.0197°/h, and the bias stability is 0.1076°/h.

The least-squares method based on the resonant frequency was used to predict the zero-rate output bias of the HRG. The results show that in the short term, the predicted bias is highly consistent with the actual output, thus effectively compensating for the gyroscope’s bias. However, from the overall fitting data, the prediction accuracy of some data fails to meet expectations. After investigating the reasons, it was found that during the temperature change process, especially in the two different stages of temperature rising and falling, the output responses of the gyroscope are not completely symmetric (hysteresis effect). This asymmetry leads to the deviation between the predicted values and the actual values.

Therefore, to further improve the accuracy of the prediction results, it is necessary to consider the non-linear characteristics of the influence of temperature changes on the gyroscope output and explore more complex models to capture this asymmetry. A new-type KAN network is adopted to train the prediction model, and multi-time series data are used for feature input in order to capture the dynamic characteristics during the process of time change.

**Figure 9:**
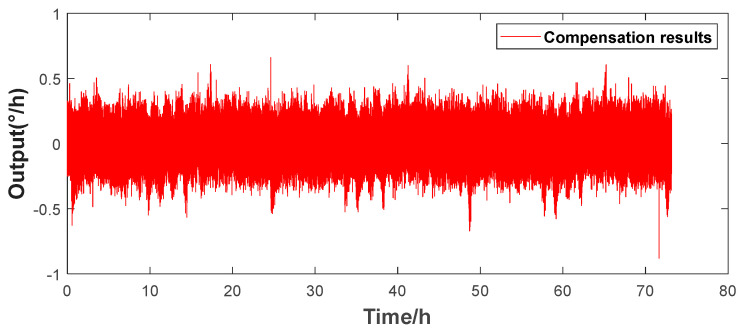
Angular rate output after compensation by the KAN prediction model.

Furthermore, the well-trained prediction model is used to predict the subsequent output of the HRG. As shown in the compensation results in Figure 9, the final bias instability is 0.013°/h, and the bias stability is 0.0651°/h. Compared with the previous two schemes, the output performance of the gyroscope has been significantly improved, effectively demonstrating the generalization ability of the KAN neural network in practical applications and verifying its effectiveness and accuracy in predicting the gyroscope output.

The results indicate that the prediction model trained by the KAN network shows remarkable effectiveness in the zero-rate output bias compensation experiment. Especially after introducing time series data as feature inputs, the model’s ability to handle the hysteresis phenomenon has been enhanced.

**Figure 10:**
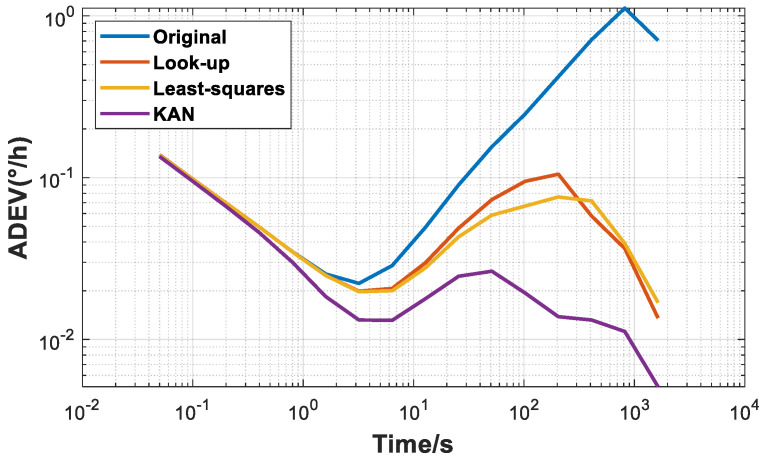
Comparison of bias instability before and after compensation of three schemes.

The above compensation results show that compared with the variation trend of the gyroscope’s zero-rate output bias before temperature compensation, all three schemes can significantly improve the output stability of the gyroscope in a temperature-varying environment, eliminating the obvious variation trend observed before compensation. The bias stabilities of the three temperature-compensated schemes are presented in Table 1 and Figure 10.

Within the temperature range of −20 °C to 60 °C, the long-term stability of the gyroscope’s output after zero-rate output bias compensation has been greatly enhanced. Specifically, in comparison with the improved look-up table method and the least-squares fitting method, the KAN neural network scheme exhibits the most outstanding compensation performance. After applying this scheme, the bias instability of the gyroscope is reduced to 0.013°/h, and the bias stability reaches 0.0651°/h, representing an approximately 40% improvement over the previous two methods.

## 6. Conclusions

This paper explores the influence mechanism of temperature changes on the zero-rate output bias of the HRG and the existing hysteresis phenomenon. It points out that during the operation of the gyroscope, there is a difference between its internal operating temperature and the external environmental temperature, which has a significant impact on the establishment of the HRG prediction model.

First, based on the working principle of the gyroscope, for the first time, the internal parameters during the gyroscope’s operation, such as the resonant frequency, the amplitude of the driving signal, and the amplitude of the quadrature suppression voltage, are used as feature inputs to establish a model of the influence on the HRG’s zero-rate output bias.

Second, within the temperature range of −20 °C to 60 °C, an HRG test experiment is designed and carried out, revealing the complex relationship between the internal parameters and the internal operating temperature of the HRG, and providing crucial data support for temperature drift compensation.

Finally, combined with the KAN neural network, a real-time temperature drift compensation model is designed. This model specifically uses time series data as input features, effectively improving the prediction accuracy of the hysteresis phenomenon and thus significantly enhancing the effect of temperature drift compensation.

During the experimental phase, by comparing the improved look-up table method and the least-squares method, the superiority of the KAN network compensation model driven by internal parameters in temperature drift compensation is verified. The results show that this model reduces the bias instability of the HRG from 0.022°/h to 0.013°/h and the bias stability from 1.1392°/h to 0.0651°/h. This indicates that the non-linear generalization ability of this model has high applicability and engineering application value for improving the long-term stability of the gyroscope’s output, providing new perspectives and solutions for research and practice in this field.

## Figures and Tables

**Table 1:** Comparison table of gyro bias stability of three schemes.

	Bias Instability	Bias Stability
Original	0.022°/h	1.1392°/h
Look-up	0.0199°/h	0.1312°/h
Least-squares	0.0197°/h	0.1076°/h
KAN	0.013°/h	0.0651°/h

## Data Availability

The data presented in this study are available on request from the corresponding author. The data are not publicly available due to privacy.
